# Protein-Losing Enteropathy Caused by Ascending Colon Cancer without Polyposis: A Rare Case Diagnosed by Scintigraphy

**DOI:** 10.70352/scrj.cr.25-0601

**Published:** 2025-12-27

**Authors:** Hisanori Miki, Takumi Yamamoto, Yusuke Kitagawa, Jun Watanabe, Yosuke Fukunaga

**Affiliations:** 1 Department of Colorectal Surgery, Kansai Medical University Medical Center, Moriguchi, Osaka, Japan; 2 Department of Colorectal Surgery, Kansai Medical University Hospital, Hirakata, Osaka, Japan

**Keywords:** protein-losing enteropathy, colon cancer, hypoalbuminemia

## Abstract

**INTRODUCTION:**

We report a rare case of protein-losing enteropathy (PLE) caused by ascending colon cancer without associated polyposis, a clinical entity that has been scarcely documented in the literature.

**CASE PRESENTATION:**

A 75-year-old man presented with bilateral lower-limb edema and cellulitis, with symptoms that had gradually progressed over 1 month. Laboratory tests revealed severe hypoalbuminemia (albumin 1.2 g/dL) and elevated carbohydrate antigen 19-9 levels, while his renal and hepatic functions were preserved, excluding other common etiologies of hypoalbuminemia. Contrast-enhanced abdominal CT demonstrated a large intraluminal tumor (58 × 78 mm) in the ascending colon, and colonoscopy confirmed a nodular obstructing lesion histologically diagnosed as well-differentiated adenocarcinoma. No evidence of diffuse or syndromic polyposis was observed. Importantly, ^99m^Tc-labeled human serum albumin scintigraphy demonstrated focal protein leakage at the tumor site, providing strong evidence that malignancy was the direct cause of PLE. Because of profound hypoalbuminemia, a robotic right hemicolectomy was performed with the creation of a double-barreled ileostomy to minimize the risk of anastomotic leakage. The final pathological diagnosis was pT3N0M0 Stage IIa well-differentiated adenocarcinoma. The postoperative course was notable for a gradual recovery of the serum albumin level, which reached 4.0 g/dL within 4 months. Concomitantly, the peripheral edema and cellulitis resolved, reflecting the reversal of the protein-losing state. The ileostomy was successfully closed 116 days after the primary surgery, and the patient remained recurrence-free with stable serum protein levels at the 5-month follow-up examination.

**CONCLUSIONS:**

To our knowledge, this is one of the few reported cases of PLE solely attributable to non-polyposis colon cancer. This case emphasizes the importance of considering PLE in the differential diagnosis of unexplained edema and hypoalbuminemia in cancer patients and highlights the diagnostic utility of albumin scintigraphy in preoperative evaluation. Furthermore, it demonstrates that prompt surgical resection can lead not only to oncological cure but also to metabolic recovery, ultimately allowing the restoration of intestinal continuity.

## Abbreviations

CA19-9
: carbohydrate antigen 19-9
CEA
: carcinoembryonic antigen
CRP
: C-reactive protein
Hb
: hemoglobin
HSA
: human serum albumin
PLE
: protein-losing enteropathy

## INTRODUCTION

PLE is a rare clinical syndrome characterized by excessive loss of plasma proteins into the gastrointestinal tract, leading to hypoalbuminemia, peripheral edema, ascites, and malnutrition in the absence of other causes, such as liver disease, nephrotic syndrome, or malabsorption disorders.^1,2)^ The pathogenesis of PLE is multifactorial and includes increased mucosal permeability, lymphatic obstruction, and structural damage to the intestinal wall due to inflammatory or neoplastic processes.^3)^

While PLE is most commonly associated with diseases such as inflammatory bowel disease, intestinal lymphangiectasia, and gastrointestinal lymphoma, its occurrence in association with colorectal neoplasms is exceedingly rare.^4,5)^ Only a handful of case reports have described PLE resulting from colorectal tumors, and most of these involved syndromic or diffuse polyposis (e.g., familial adenomatous polyposis or cap polyposis).^6–8)^ In contrast, PLE caused solely by sporadic colorectal carcinoma without polyposis has rarely been reported in the relevant literature.

We herein present a unique case of PLE caused by non-polypoid ascending colon cancer in a patient without underlying hereditary polyposis. The diagnosis of PLE in this case was confirmed using ^99m^Tc-labeled HSA scintigraphy, and the patient showed prompt clinical and biochemical improvement following surgical resection. This case underscores the importance of considering PLE in patients with unexplained hypoalbuminemia and highlights the diagnostic utility of scintigraphy in identifying protein leakage from gastrointestinal malignancies.

## CASE PRESENTATION

A 75-year-old man presented with progressive bilateral lower-limb edema and cellulitis of the left leg and was referred to our hospital. He had first noticed leg swelling approximately 1 month prior to admission. His medical history included hypertension, hyperlipidemia, and a remote appendectomy. His laboratory data revealed severe hypoalbuminemia (serum albumin, 1.2 g/dL; total protein, 3.8 g/dL), mild anemia (Hb, 10.2 g/dL), and elevated CRP (7.3 mg/dL). His serum CEA level was within the normal range (3.8 ng/mL), whereas CA19-9 was mildly elevated at 47.5 U/mL. There was no proteinuria, no signs of liver dysfunction were observed, and his nutritional intake was adequate, suggesting a gastrointestinal source of protein loss.

**Fig. 1:**
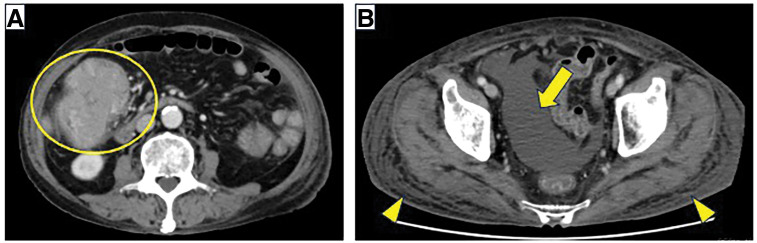
Contrast-enhanced abdominal CT. (**A**) A 58 × 78-mm enhancing mass lesion is observed in the ascending colon (yellow circle). (**B**) Ascites (arrow) and subcutaneous edema (arrowhead) are also noted.

**Fig. 2:**
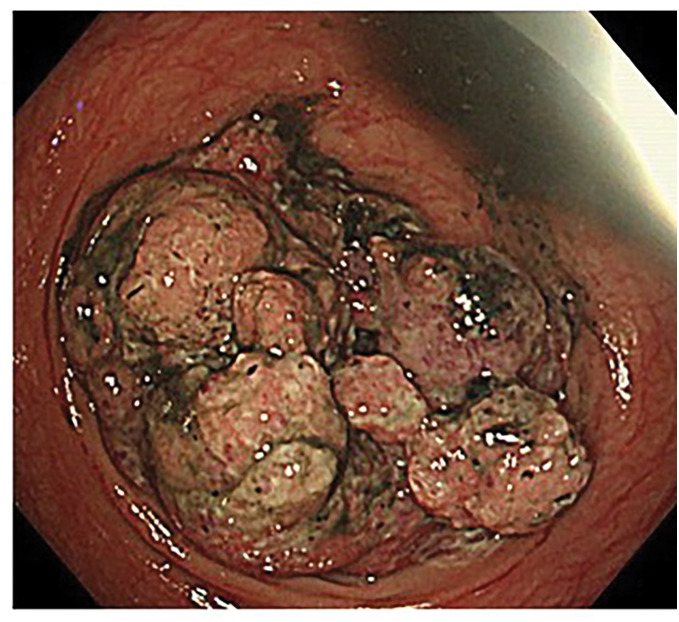
Colonoscopy. A type 1 tumor occupying the lumen of the ascending colon is observed.

Contrast-enhanced abdominal CT revealed a 58 × 78 mm mass with heterogeneous enhancement in the ascending colon (**Fig. 1A**), accompanied by subcutaneous edema and ascites (**Fig. 1B**). Colonoscopy revealed a large, nodular obstructing tumor in the ascending colon (**Fig. 2**). Biopsy specimens showed a well-differentiated adenocarcinoma.

**Fig. 3:**
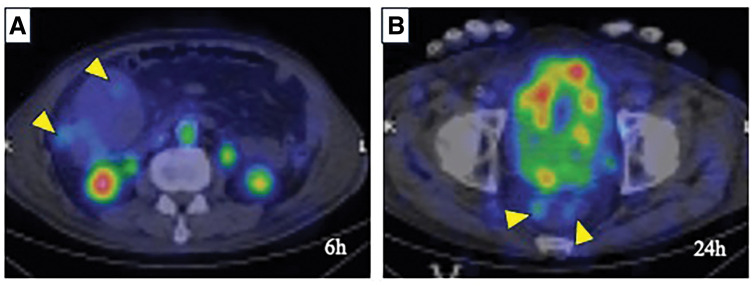
^99m^Tc-labeled HSA scintigraphy. (**A**) At 6 h, protein leakage is observed within the tumor (arrowhead). (**B**) At 24 h, the leaked protein is seen flowing into the rectum (arrowhead). HSA, human serum albumin

To evaluate PLE, ^99m^Tc-labeled HSA scintigraphy showed faint accumulation at the tumor site (**Fig. 3**), consistent with localized protein leakage.

**Fig. 4:**
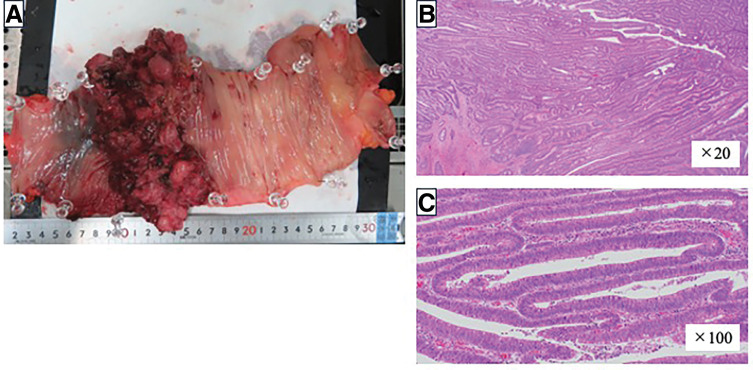
Histopathological findings. (**A**) The resected specimen shows a circumferential, elevated lesion in the ascending colon without features of polyposis. (**B**) Tubulovillous adenocarcinoma (×20 magnification). No lymphatic dilatation was observed. (**C**) Tubulovillous adenocarcinoma (×100 magnification). No lymphatic dilatation was observed.

On the basis of these findings, the patient was diagnosed with ascending colon cancer complicated by PLE. He underwent robot-assisted right hemicolectomy without anastomosis because of concerns about anastomotic healing under profound hypoalbuminemia. A double-barreled ileostomy was performed. The operative time was 276 min, and the intraoperative blood loss was 1179 mL. Intraoperatively, a moderate amount of serous, light-yellow ascitic fluid was observed. Additionally, there was bleeding from an injury to the accessory right colic vein, which contributed to the total fluid volume of 1179 mL. Therefore, the reported value includes both ascitic fluid and blood loss, resulting in a larger total volume than in typical right hemicolectomies. A histopathological analysis revealed a pT3 N0 M0 (Stage IIa) well-differentiated adenocarcinoma with partial papillary features (**Fig. 4**).

Postoperatively, the patient’s serum albumin level began to increase promptly, reaching 1.9 g/dL on POD 20 and 2.6 g/dL by POD 39. His lower-limb edema and cellulitis concurrently improved. The patient was discharged on POD 20. On POD 115, his serum albumin level had normalized to 4.0 g/dL, and stoma closure was performed on POD 116. The patient remained recurrence-free and normoalbuminemic, without any further symptoms of edema on POD 154.

## DISCUSSION

PLE is a rare but potentially debilitating condition characterized by the abnormal loss of serum proteins into the gastrointestinal tract, leading to hypoalbuminemia, edema, and immunodeficiency.^1,3)^ While PLE is more commonly associated with conditions such as intestinal lymphangiectasia, inflammatory bowel disease, and autoimmune enteropathies,^2,9)^ its association with colorectal cancer is exceedingly rare and poorly understood. To our knowledge, only a handful of case reports have described PLE secondary to colorectal tumors, most of which involved diffuse polyposis syndromes (e.g., familial adenomatous polyposis or cap polyposis).^5–7)^

In contrast, our patient exhibited PLE caused by solitary and sporadic ascending colon cancer in the absence of polyposis or syndromic features. This case is noteworthy not only for the rarity of its etiology but also for the preoperative confirmation of protein leakage via ^99m^Tc-labeled HSA scintigraphy. While α1-antitrypsin clearance remains a conventional diagnostic tool for PLE, scintigraphy has emerged as a noninvasive modality capable of localizing the site of protein leakage.^10,11)^ In our case, the faint but localized uptake corresponding to the tumor supported the diagnosis of cancer-induced PLE and helped to guide surgical planning.

The pathophysiological mechanism by which colorectal cancer causes PLE has not been fully elucidated. The proposed mechanisms include increased mucosal permeability due to tumor invasion, lymphatic obstruction, and direct leakage of plasma proteins through ulcerated tumor surfaces.^2,4)^ Nomura et al.^4)^ reported marked lymphatic dilatation within the tumor specimen and discussed that elevated lymphatic pressure at the tumor surface might lead to protein leakage. In contrast, our case showed no abnormal lymphatic dilatation within the specimen, suggesting that protein leakage occurred via a different mechanism. This finding suggests that tumor-associated microvascular or lymphatic changes may have caused the protein loss.

We administered 25 g of HSA daily for 4 consecutive days before surgery. However, the serum albumin level on the morning of surgery remained low at 1.9 g/dL. Despite albumin supplementation, continuous protein loss prevented sufficient improvement. Therefore, considering the high risk of anastomotic leakage due to persistent hypoalbuminemia, we decided to perform a diverting stoma instead of primary anastomosis. Importantly, surgical resection of the tumor led to rapid clinical improvement. Serum albumin levels began to rise immediately after surgery and normalized within 2 months. Concurrently, the patient’s lower-limb edema and cellulitis resolved. The patient’s recovery was sufficiently robust to permit the reversal of the double-barreled ileostomy by POD 80. These outcomes underscore the curative potential of surgical management in appropriately selected patients with tumor-associated PLE.

This case report highlights several clinically relevant issues. First, PLE should be considered in the differential diagnosis of unexplained hypoalbuminemia and edema, particularly when common causes, such as liver disease, nephrotic syndrome, or malnutrition, have been excluded. Second, nuclear scintigraphy is a useful adjunctive diagnostic tool for localizing and supporting the diagnosis of gastrointestinal protein loss, particularly in malignancy-associated PLE. Finally, prompt tumor resection may not only achieve oncological control but also resolve the metabolic consequences of protein loss, thereby potentially allowing complete surgical restoration, as was achieved in this case.

Given the rarity of colorectal cancer-associated PLE, further case accumulation and mechanistic studies are needed to clarify its pathophysiology and inform management strategies.

## CONCLUSIONS

This case highlights the rare occurrence of PLE as a paraneoplastic manifestation of non-polypotic colon cancer. The preoperative diagnosis of PLE was successfully achieved using ^99m^Tc-labeled HMA scintigraphy, which localized the site of protein loss to the tumor. Surgical resection of the cancer resulted in rapid normalization of the patient’s serum albumin level, resolution of peripheral edema, and, ultimately, successful closure of the diverting ileostomy. This case emphasizes the importance of considering PLE in the differential diagnosis of unexplained hypoalbuminemia and demonstrates that timely oncologic surgery can be both diagnostic and curative in selected patients with malignancy-associated PLE.

## DECLARATIONS

### Availability of data and materials

All data generated or analyzed during this study are included in this article. Further inquiries may be directed to the corresponding authors.
